# Human papillomavirus, high-grade intraepithelial neoplasia and killer immunoglogulin-like receptors: a Western Australian cohort study

**DOI:** 10.1186/1750-9378-8-33

**Published:** 2013-09-06

**Authors:** Brian Brestovac, Michelle E Wong, Raymond Tjendera, Paul J Costantino, Cyril Mamotte, Campbell S Witt

**Affiliations:** 1 School of Biomedical Sciences, CHIRI Biosciences Research Precinct, Faculty of Health Sciences, Curtin University, GPO Box U1987, Perth, Western Australia 6845, Australia; 2 Department of Clinical Immunology and Immunogenetics, Royal Perth Hospital, Western Australia, Australia

**Keywords:** Cervical cancer, High-grade CIN, KIR, Natural killer cells, Human papillomavirus

## Abstract

**Background:**

Human papillomavirus (HPV) is the causative agent in cervical cancer and HPV genotypes 16 and 18 cause the majority of these cancers. Natural killer (NK) cells destroy virally infected and tumour cells via killer immunoglobulin-like receptors (KIR) that recognize decreased MHC class I expression. These NK cells may contribute to clearance of HPV infected and/or dysplastic cells, however since KIR controls NK cell activity, KIR gene variation may determine outcome of infection.

**Methods:**

KIR gene frequencies were compared between 147 patients with a history of high-grade cervical intraepithelial neoplasia (CIN) and a control population of 187, to determine if any KIR genes are associated with high-grade CIN. In addition a comparison was also made between cases of high grade CIN derived from 30 patients infected with HPV 16/18 and 29 patients infected with non-16/18 HPV to determine if KIR variation contributes to the disproportional carcinogenesis derived from HPV 16/18 infection.

**Results:**

High-grade CIN was weakly associated with the absence of KIR2DL2 and KIR2DS2 (p = 0.046 and 0.049 respectively, OR 0.6; 95% CI 0.4 – 0.9) but this association was lost after correction for multi-gene statistical analysis. No difference in KIR gene frequencies was found between high-grade CIN caused by HPV 16/18 and non-16/18.

**Conclusion:**

No strong association between KIR genes, high-grade CIN and HPV genotype was found in the Western Australian population.

## Background

Cervical cancer is the second most prevalent cancer in women worldwide accounting for approximately 230 000 deaths each year and Human Papillomavirus (HPV) is the causative agent [1]. HPV genotypes 16 and 18 cause the majority of cancers and it was reported that approximately 80% of cervical cancers and 50% of high-grade pre-cancerous lesions in Western Australia are caused by these two genotypes [2]. Progression to cancer is preceded by pre-cancerous lesions; the majority of low-grade lesions, cervical intraepithelial neoplasia 1 (CIN 1) regress spontaneously, while high-grade lesions (CIN 2 and CIN 3) often persist and may progress to cancer [3,4]. It is thought that the immunological response may affect the progression of cervical lesions and in particular, that natural killer (NK) cells may play a role in preventing disease development [5-8].

Many viruses commonly down-regulate major histocompatibility complex (MHC) class I molecule expression to avoid cytotoxic T cell responses and the down-regulation of MHC class I molecules has been a common finding in cervical cancer [9-12]. It has been found that the HPV E5 gene encodes a protein that causes the alkalinisation of the golgi apparatus and endosomes inducing the retention of MHC class I complexes, thereby preventing their transport to the cell surface [13]. It has also been reported that the HPV E7 gene codes for a protein that interrupts the synthesis of MHC class I molecules [14].

NK cells are cytotoxic lymphocytes which kill target cells with decreased MHC class I molecule expression as a defence mechanism against malignant transformation or viral infection [15,16]. Previous studies which have investigated the influence of NK cells in CIN development have suggested a protective role of NK cells. An early report found defective NK cell lysis of HPV 16 infected keratinocytes in patients with CIN or cancer [17]. In another study NK cells isolated from patients with advanced cervical cancer demonstrated lower levels of cytotoxicity against the human erythroleukaemia K562 cell line than NK cells from women without cervical cancer suggesting that NK cell activity was inversely correlated with severity of disease [7]. Human NK cell activity is controlled by the expression of activating and inhibitory receptors which recognise ubiquitously expressed MHC class I molecules. Killer immunoglobulin-like receptors (KIR) are genetically highly polymorphic and recognize polymorphic HLA epitopes [18]. The KIR gene family is found on chromosome 19 and encodes receptors with either two (2D) or three (3D) extracellular immunoglobulin-like domains. Inhibitory receptors possess a long (L) cytoplasmic tail and activating receptors possess a short (S) cytoplasmic tail [19,20]. Individuals inherit different numbers and types of KIR genes [21]. Generally, the KIR3D receptors recognise HLA-A and HLA-B alleles, while KIR2D receptors recognise HLA-C alleles [22]. KIR diversity has been driven by rapid evolution to a variety of forces including response to pathogens [20]. While determining genetic links to cervical cancer have been elusive [23] recent studies have suggested an association between KIR gene repertoire and cervical cancer [24,25], however these studies examined different populations (US/Costa Rica and Sweden) and reported associations with different KIR genes.

The current study was undertaken to determine whether there are any associations between KIR genes and high-grade cervical intraepithelial neoplasia (CIN2 & 3), the precursor to cervical cancer, in the Western Australian population. Since 80% of cervical cancers in Western Australia are caused by HPV 16 and 18 genotypes (HPV 16/18), we performed a separate analysis of high-grade CIN samples containing these genotypes on the basis that neoplasia derived from these may be especially resistant to NK cell killing.

## Results

**Figure 1:**
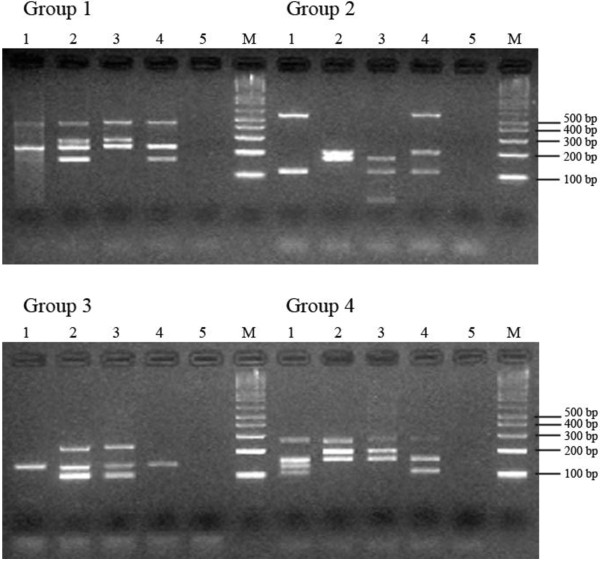
**Examples of KIR genotyping results using cervical cytology samples.** The KIR genotyping result for each sample was determined by comparing the band sizes to the molecular weight marker. For Group 1, Group 2, Group 3 and Group 4; lanes 1 and 2 are JBUSH and CB6B genomic DNA positive controls respectively, lanes 3 and 4 are the KIR PCR products from DNA extracts of cervical cytology specimens and lane 5 is the negative control. Lane M is the molecular weight marker. Clear PCR products for KIR typing were achieved with cervical cytology samples seen in lanes 3 and 4.

KIR genotyping was successfully performed on all cervical cytology specimens. Figure 1 show that the band intensity of PCR products from DNA templates extracted from cytological cervical ThinPrep samples in lanes 3 and 4 were comparable to those of the standards in lanes 1 and 2 which were extracted from cell lines (Figure 1).

All the KIR genes were represented in both the high grade CIN cases and controls. Table 1 compares the frequencies of each KIR gene in cases and controls. KIR2DL2 and KIR2DS2 frequencies were significantly decreased in the high-grade CIN cases compared to the controls (p = 0.046 and 0.049 respectively, OR 0.6; 95%CI 0.6 – 0.9). However, significance was lost after Bonferroni correction (p = 0.644 and p = 0.686, respectively). No statistically significant differences were found for any of the remaining KIR gene frequencies. There was also no significant difference between patients and controls for the total numbers of either activating or inhibitory KIR (activating p = 0.47, inhibitory p = 0.45), nor any significant differences in any KIR gene frequencies between the high grade CIN samples derived from HPV 16/18 infection and those that were derived from non-16/18 infection (Table 2).

**Table 1:** Distribution of KIR genes in the Busselton control population and the group of Western Australian women with a history of CIN

***KIR *gene**	**Cases**	**Controls**	**p-value**^*a*^	**p-value corrected**^*b*^
	**n = 147**	**n = 187**		
***2DL1***	144 (98.0%)	178 (94.2%)	0.149	1
***2DL2***	67 (45.6%)	108 (57.1%)	0.046*	0.644
***2DL3***	136 (92.5%)	170 (89.9%)	0.531	1
***2DL4***	147 (100%)	189 (100%)	1	1
***2DL5***	78 (53.1%)	100 (52.9%)	0.978	1
***3DL1***	133 (90.5%)	177 (93.7%)	0.382	1
***3DL2***	147 (100%)	189 (100%)	1	1
***3DL3***	147 (100%)	189 (100%)	1	1
***2DS1***	58 (39.5%)	76 (40.2%)	0.978	1
***2DS2***	68 (46.3%)	109 (57.7%)	0.049^*^	0.686
***2DS3***	44 (29.9%)	55 (29.1%)	0.964	1
2DS4^**^	55 (37.4%)	77 (40.7%)	0.612	1
***2DS5***	50 (34.0%)	57 (30.2%)	0.526	1
***3DS1***	61 (41.5%)	78 (41.3%)	0.967	1

CIN, Cervical intraepithelial neoplasia; KIR, Killer immunoglobulin-like receptor.

^*a*^*χ*^2^ test with Yates correction was performed; p-values < 0.05 are indicated in bold for *χ*^2^ test.

^*b*^Corrected p-values were determined by adjusting p-values for multi-gene analysis using Bonferroni’s correction.

^*^OR = 0.6; 95% CI 0.4 – 0.9.

^**^2DS4 psuedogenes not counted.

**Table 2:** Distribution of KIR genes in HPV 16/18 and non 16/18 derived high grade CIN

***KIR*gene**	**HPV 16/18**	**Non 16/18**	**p-value**^*a*^
	**n = 30**	**n = 29**	
***2DL1***	28 (93%)	27 (93%)	0.97
***2DL2***	12 (40%)	16 (55%)	0.36
***2DL3***	23 (74%)	21 (72%)	0.87
***2DL4***	30 (100%)	29 (100%)	1
***2DL5***	12 (39%)	17 (59%)	0.20
***3DL1***	29 (96%)	24 (83%)	0.10
***3DL2***	30 (100%)	29 (100%)	1
***3DL3***	30 (100%)	29 (100%)	1
***2DS1***	12 (39%)	14 (48%)	0.63
***2DS2***	15 (48%)	18 (62%)	0.42
***2DS3***	7 (22%)	6 (21%)	0.86
***2DS4***	25 (81%)	21 (72%)	0.65
***2DS5***	12 (39%)	13 (45%)	0.83
***3DS1***	12 (39%)	13 (45%)	0.83

HPV, Human Papillomavirus; KIR, Killer immunoglobulin-like receptor.

^*a*^*χ*^2^ test with Yates correction was performed; p-values < 0.05 for significance.

## Discussion

Risk factors which determine susceptibility to HPV infection and progression to cervical cancer are likely to be immunogenetic [8,23] and this is supported by the high prevalence of cervical lesions in immunosuppressed patients [6]. Population-based studies in the United States and in Costa Rica reported KIR3DS1 to be associated with an increased risk for developing high-grade CIN or cervical cancer, however they only considered CIN 3 as high-grade and CIN 2 was not included [24]. In the current study, there was no significant difference between the control and cases in KIR3DS1 frequency. Also, in this study both CIN 2 and 3 were considered as high-grade CIN. Arnheim et al. [25] did not find any single gene to be associated with CIN but found that a genotype comprising only KIR2DL1, KIR2DL2, KIR2DL3, KIR2DL4, KIR3DL1, KIR3DL2, KIR3DL3 and KIR2DS4 was associated with an increased risk of developing CIN. The same study reported that KIR2DL5 was decreased in patients with CIN. However this previous study was based on all stages of CIN, and it is known that the majority of low-grade lesions (CIN1) regress spontaneously [2,4,26]. The inclusion of low-grade lesions in any study as a precursor to cervical cancer remains controversial [26]. In the current study no association with high-grade CIN (CIN 2 & 3) was found with KIR3DS1 or KIR2DL5. Indeed the frequency of these two genes was virtually identical in patients and controls (Table 1). We also did not find any cases or controls with a genotype comprising only KIR2DL1, KIR2DL2, KIR2DL3, KIR2DL4, KIR3DL1, KIR3DL2, KIR3DL3 and KIR2DS4 as reported by Arnheim et al. [25]. Such a genotype which includes KIR2DL2 and not KIR2DS2 would be very rare in any population as these two genes are usually in complete linkage disequilibrium [27]. The only association found in the current study was a protective effect of KIR2DL2 and KIR2DS2 which was not significant after correction for multiple gene analysis. This finding does not support the study of Carrington et al. [24] in which these two genes were more prevalent in cases than controls, though not significantly so.

Although HPV 16 and 18 cause the majority of cervical cancers in Western Australia, this study found no statistical significant difference in any KIR gene frequency between HPV 16/18 and non-16/18 derived high grade CIN. The protective effect of KIR2DL2 and KIR2DS2 was not more pronounced in the cases that were positive for the more oncogenic HPV 16/18 types of virus. However, since HPV has been associated with some head and neck cancers [28], and the pathogenesis for these cancers is different, there would be value in examining the association between these and KIR.

## Conclusion

This study found no strong association between KIR gene frequencies and high-grade CIN in the Western Australian population, although KIR2DL2 and KIR2DS2 may have a very mild protective role against progression to high grade CIN. No association between KIR gene frequencies and HPV 16/18 derived high grade CIN was found.

## Methods

### Study population

Cervical cytology specimens in ThinPrep (Cytyc, Boxborough, MA) methanol-based fixative, were collected between December 2007 and August 2008 from Western Diagnostics Pathology Laboratory, Perth, Western Australia. The study population consisted of 147 women from Western Australia who had previously been diagnosed with high-grade CIN. This test group had a mean age of 38 with a range of 19 – 71 years. The control frequencies for KIR genes were obtained from 187 women from the Busselton Health Study (BHS) (http://bsn.uwa.edu.au/) population which is considered to be reflective of the Western Australian population. This control group had a mean age of 50 with a range of 19 – 88 years. Although CIN status was not obtained for this control group high-grade CIN occurs very rarely [29] and so would not impact on the statistics. Power calculations indicate that this study had approximately 80% power to detect an odds ratio (OR) of less than 0.5 or greater than 2.0. High-grade CIN ThinPrep samples, for which the HPV genotype had previously been determined by PCR and DNA sequencing in the L1 region [2], were further grouped into those derived from HPV 16/18 (n = 30) infections and those from non-16/18 (n = 29). This study was approved by the Human Research Ethics Committee at Curtin University of Technology (approval number SoBS08/07).

### DNA extraction and PCR

Genomic DNA was extracted from cytology specimens by a modified EDNA HiSpEx™ tissue kit (Saturn Biotech, Perth, Australia) method in which ThinPrep cervical samples were mixed by shaking and inversion, a 500 μl aliquot was removed and mixed with 1 mL of PCR grade water in a microfuge tube. This was centrifuged at 14,000 RPM for 5 minutes and all traces of the supernatant removed from the pellet. The pellet was then processed according to the manufactures protocol. It is recognized that cells containing integrated HPV would have genomic alterations; however the majority of cells within the ThinPrep sample would not be infected with HPV and would have normal genomes, and so were considered suitable for KIR typing by PCR.

KIR genotyping was performed on all samples using multiplexed amplification primers specific for the following KIR genes: 2DL1, 2DL2, 2DL3, 2DL4, 2DL5, 2DS1, 2DS2, 2DS3, 2DS4, 2DS5, 3DL1, 3DL2, 3DL3 and 3DS1 as described by Sun et al. [29]. In each typing run 10th International Histocompatibility Workshop cell lines, CB6B and JBUSH, were used as positive controls [30]. PCR products were electrophoresed on a 2% agarose gel in 1× TAE buffer precast in SYBR Safe (Invitrogen, Carlsbad, CA) and photographed under a UV transilluminator.

### Statistical analysis

KIR gene frequencies in high grade CIN cases and controls, and in HPV 16/18 and non 16/18 derived high grade CIN, were compared by *χ*^2^ analysis with Yates’ correction. A p-value less than 0.05 determined significance. Any p values less than 0.05 were corrected for multi-gene statistical analysis using Bonferroni’s correction [31].

## Abbreviations

HPV: Human papillomavirus; CIN: Cervical intra-epithelial neoplasia; KIR: Killer immunoglobulin-like receptor; NK: Natural killer; MHC: Major histocompatibility complex; HLA: Human leukocyte antigen; BHS: Busselton health study; PCR: Polymerase chain reaction; OR: Odds ratio; CI: Confidence interval.

## Competing interests

The authors declare that they have no competing interests.

## Authors’ information

All authors are from the School of Biomedical Sciences at Curtin University except CW who is a senior research scientist at the Dept of Clinical Immunology and Immunogenetics, Royal Perth Hospital, Western Australia.

## Authors’ contributions

BB, PC, CM and CW participated in the design of the study and performed analysis of data. MW performed sample extraction, KIR typing for the KIR vs CIN section and contributed to the analysis of that data. RT performed sample extraction, KIR typing for the HPV 16/18 vs non 16/18 section and analysis of that data. All authors read and approved the final manuscript.
